# Autosensitization Triggered by Neosporin Use: A Unique Phenomenon

**DOI:** 10.7759/cureus.26987

**Published:** 2022-07-18

**Authors:** Jasdeep S Bathla, Komalpreet Tur, Zara Sragi, Kyle Sugg

**Affiliations:** 1 Internal Medicine, Wayne State University Detroit Medical Center, Detroit, USA; 2 Internal Medicine, Wayne State University School of Medicine, Detroit, USA

**Keywords:** drug-induced hypersensitivity, hypersensitivity, id reaction, neosporin, secondary dermatosis, allergic contact dermatitis, autosensitization, interface dermatitis, autoeczematization

## Abstract

Interface dermatitis is a type of dermatological insult to the dermo-epidermal skin junction. When this reaction causes secondary dermatosis that occurs distally on the body, it is known as autosensitization or autoeczematization. Here, we present the case of a middle-aged gentleman, with a medical history including human immunodeficiency virus, non-adherent to highly active antiretroviral therapy, initially presenting due to chronic recurring cellulitis on the left lower leg that had become progressively erythematous and tender to palpation. A few days later, he developed an intensely pruritic, papular rash on his face and chest. Following further investigation, he reported using Neosporin ointment on the leg rash prior to admission which had then caused allergic contact dermatitis. Ultimately, the patient was diagnosed with acute interface dermatitis due to Neosporin use, which led to a secondary autosensitization reaction involving his head, neck, and arms. This case illustrates the importance of thorough history taking and clinical suspicion to appropriately diagnose this phenomenon, further demonstrating the temporal association between allergic contact dermatitis and autosensitization.

## Introduction

Interface dermatitis is a dermatological reaction that occurs following damage to the interface or the dermo-epidermal skin junction. Common interface dermatitides include lichen planus, erythema multiforme, and several other inflammatory, infectious, and even neoplastic skin changes. This injury can lead to secondary dermatitis found distally on the body away from the area of the initial insult. This process is known as an autosensitization or autoeczematization reaction and represents autoimmune pathology that usually develops a few days to weeks after the primary trigger. While the exact incidence of these secondary reactions is not yet established, the most common causes include chronic venous stasis dermatitis and superficial fungal infections classically with tinea pedis or other dermatophytes that can cause an eruption of lesions on the hands or legs. Other lesser reported cases include post-immunization reactions (most notably with the BCG vaccine) and following administration of immunotherapy. Medications present a much less common etiology for this rash; however, its onset appears to be related to the onset of drug-induced allergic contact dermatitis.

## Case presentation

This is a case of a 39-year-old male with a medical history significant for human immunodeficiency virus (HIV), not adherent to highly active antiretroviral therapy (HAART), disseminated and untreated varicella-zoster virus (VZV) infection, untreated hepatitis B, and recurrent cellulitis of the left lower leg following a motor vehicle accident two years prior to this hospitalization for which he required a left lateral leg fasciotomy. He presented to our emergency department with primary complaints of a progressive, vesicular, and erythematous rash on the lateral aspect of the lower left leg that first appeared approximately one week prior and had recently started to spread diffusely across the entire body. The affected area of the lower left extremity progressively became swollen and pruritic and was soon followed by a “bumpy rash” that had spread over his body, most notably behind his ears bilaterally as well as into the groin region. Although the patient was non-adherent to HAART, he did demonstrate preserved CD-4 count (>500 cell/µL) about four months prior with a viral load at that time of roughly 9,000 RNA copies/mL. On further evaluation, he also endorsed recent cocaine use, as well as daily tobacco smoking.

**Figure 1:**
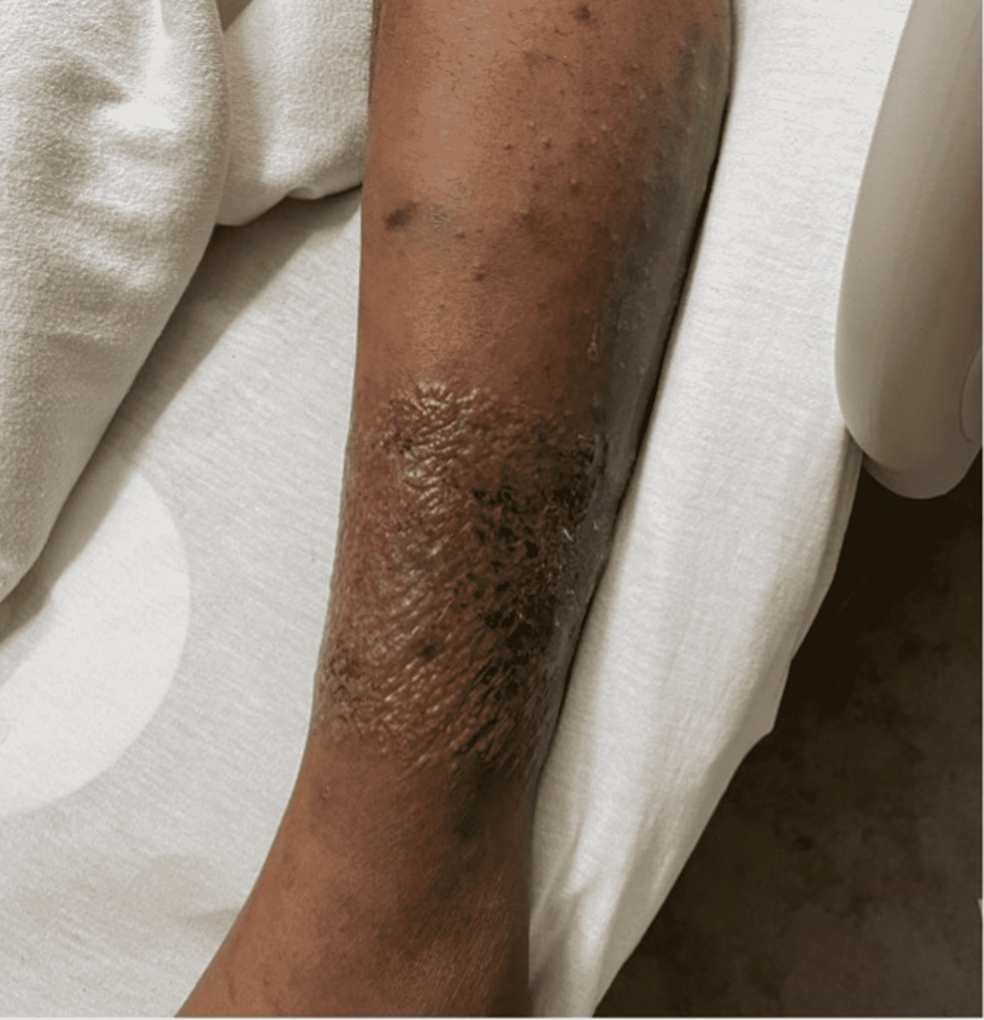
A collection of clear fluid-filled vesicles, well-demarcated with an erythematous base.

**Figure 2:**
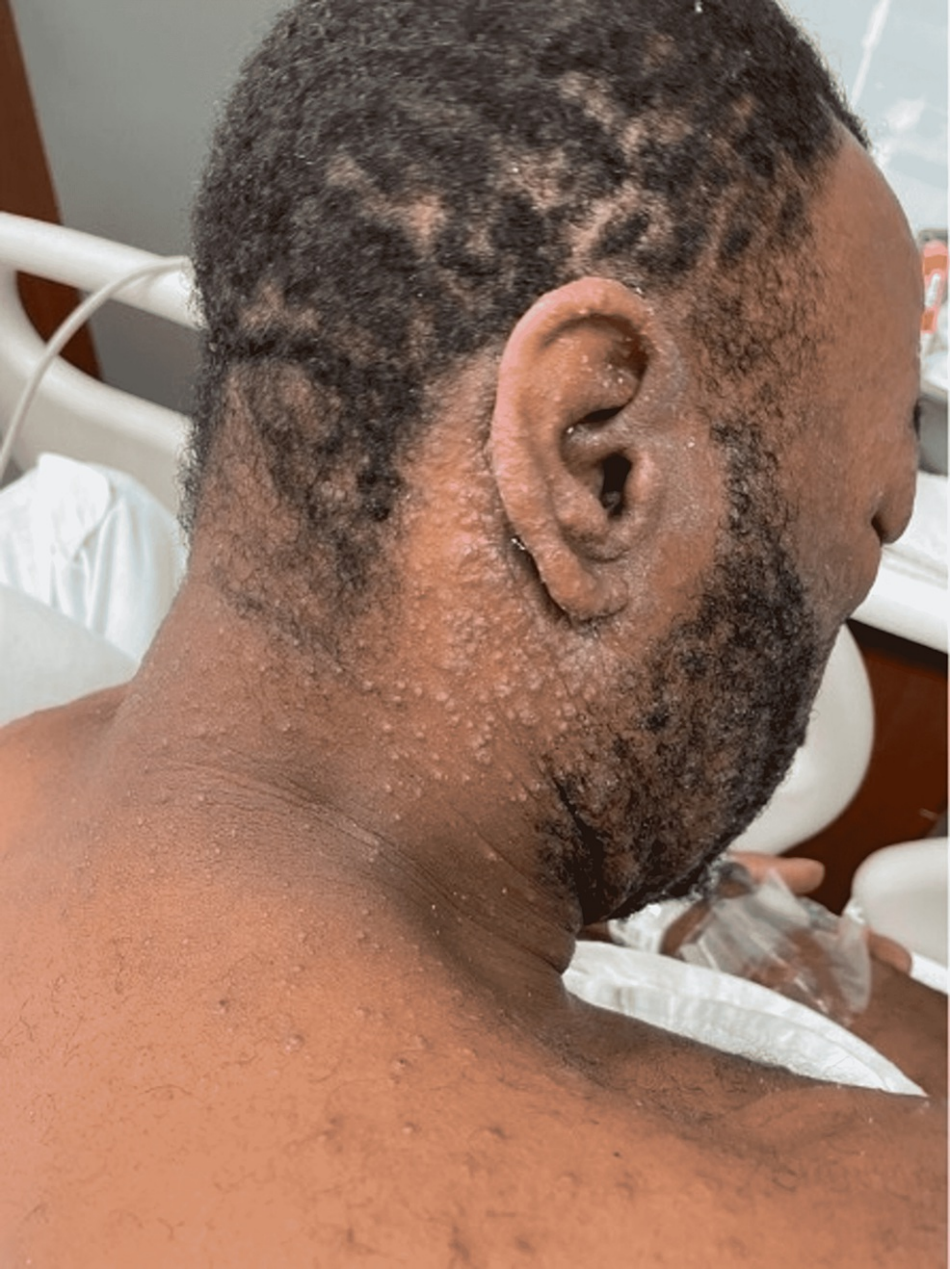
Vesicular rash of the post-auricular and nape regions.

Physical examination found a 1.5-inch scar on the left lower leg from his previous fasciotomy along with a well-demarcated, erythematous collection of clear fluid-filled vesicles present on the anterior and lateral aspects (Figure 1). Bilateral aspects of the patient’s neck were also covered with these clear vesicular lesions including the post-auricular and nape areas (Figure 2).

On the second day of admission, the patient complained of generalized whole-body pruritis associated with a progressively spreading rash over his anterior chest, upper extremities, and genitals. There was also bilateral swelling of the superficial eyelids without any drainage. Dermatology was consulted at this stage as the patient’s clinical status was not improving. On their evaluation, the patient disclosed that he had been applying Neosporin gel to the left leg rash prior to admission. It was determined that the patient’s use of Neosporin had triggered allergic contact dermatitis on the leg as evidenced by the geometric distribution of the clear vesicles in a rectangular display (Figure 1). The newer lesions present on the ears, face, chest, and upper extremities were consistent with acute interface dermatitis that arose a few days following the initial dermatosis of the left leg. Along with diphenhydramine and famotidine for symptomatic relief, the patient was also administered 0.1% triamcinolone ointment to use on all affected areas of the rash. After just a single day of topical steroid use, the patient’s dermatosis began to improve significantly. He was discharged home on an oral steroid taper, with follow-up planned in the dermatology clinic.

## Discussion

The majority of autosensitization cases occur secondary to chronic venous stasis dermatitis with an incidence rate of approximately 37% and additional cases do occur in patients following infection with microbiota, usually superficial dermatophytes [1]. In these instances, keratinocytes at the site of initial irritation produce proinflammatory cytokines, which activate local T-lymphocytes; hence, the process is called autosensitization or autoeczematization. These T-lymphocytes can then disseminate and cause a reaction at a different site of the body [2].

Autosensitization reactions may also be present in patients with a history of contact dermatitis [2]. Classically, they present as a vesicular patch on the legs, arms, and/or trunk with a surrounding area of erythema that defines the local site of eruption. Areas with a previous dermatological injury such as dermatoses, scars, and/or burns are common locations for this to occur. As the rash progresses, these lesions disseminate to form symmetric patches that are intensely pruritic [2]. As seen in our case, the initial allergic contact dermatitis developed at the site of a previous surgical scar where our patient was applying Neosporin gel. The vesicular lesions were arranged in a geometrical rectangular distribution consistent with a delayed-type hypersensitivity reaction (type IV) to exogenous stimuli.

Neosporin, usually used for superficial wound infections, is widely known to cause allergic contact reactions. Neomycin, the most bioactive component of Neosporin, is a part of the aminoglycoside antibiotic class used to treat many gram-positive and gram-negative bacterial infections. A derivative of neomycin, appropriately named neomycin B or framycetin, is used in other topical ointments, including anti-hemorrhoidal creams such as Proctosedyl and Proctomyxin. In their case series, Hughes and Pratt detailed two patients who developed allergic contact dermatitis and subsequent autosensitization reaction following the use of these creams [3]. They patch-tested the patients for allergies and found they were “strongly positive for reaction” to common aminoglycosides (namely, neomycin sulfate, gentamicin sulfate, kanamycin sulfate, and framycetin sulfate). This demonstrates the drug class as a common allergen with cross-reactivity among the various aminoglycoside components found widely in topical creams.

The process by which allergic contact dermatitis occurs can be divided into a sensitization and elicitation stage [4,5]. First, exposure to non-immunogenic haptens binds to proteins located within the superficial skin layers. Next, this protein-hapten complex is phagocytosed by local Langerhans cells, displayed on their cell surfaces, and then carried to nearby lymph nodes. Here, T-lymphocytes specific to the hapten-antigen are created, thus sensitizing the body’s immune system. Lastly, a cytokine-mediated inflammatory response due to the proliferation of activated T-lymphocytes is elicited upon re-exposure to either the pre-sensitized agent or a similar agent with cross-reactivity [4,5]. These T-lymphocytes can then spread systemically via the bloodstream to other parts of the body where they can trigger a secondary dermatological response known as an autosensitization or autoeczematization reaction [2]. Patients undergoing an autosensitization reaction may have higher levels of human leukocyte antigen-DR and interleukin-2 receptors expressed on their T-lymphocytes, with a return to normal levels upon resolution of the acute secondary reaction [2].

## Conclusions

Overall, autosensitization is a relatively rare phenomenon with few cases occurring following non-infectious etiology. Diagnosing this dermatitis can be difficult not only due to its relatively low incidence but also because the secondary rash that occurs is often attributed to an unrelated trigger or inciting factor, thereby making it harder to establish a temporal association. The mechanism of action of this secondary process demonstrates a strong relationship to an initial dermatosis, especially allergic contact dermatitis. Although the diagnosis is supported by patch testing, this is not usually feasible or practical in an acute setting. Therefore, the diagnosis is made clinically with close attention to patient history and timeline. Other differentials to be mindful of when making this diagnosis include contact dermatitis, herpetic infections, insect bite reactions, and drug eruptions. Because of the difficulty in diagnosis, accurate history and attention to key details and exposure in the timeline are vital in narrowing the differential. Although many cases are self-limited, therapy is ultimately completed by a short course of corticosteroids, antihistamines, and supportive wet compresses, as well as by addressing the underlying stimulus for the autosensitization.
